# Pharmacokinetics, safety, and tolerability of single and multiple doses of zuranolone in Japanese and White healthy subjects: A phase 1 clinical trial

**DOI:** 10.1002/npr2.12359

**Published:** 2023-06-27

**Authors:** Takuhiro Sonoyama, Ryosuke Shimizu, Ryuji Kubota, Yumiko Matsuo, Daiki Okutsu, Hideki Yamanaka, Keiko Takasu, Koichi Ogawa, Tomoko Motomiya

**Affiliations:** ^1^ Medical Science Department, Drug Development and Regulatory Science Division Shionogi & Co., Ltd. Osaka Japan; ^2^ Clinical Pharmacology & Pharmacokinetics, Drug Development and Regulatory Science Division Shionogi & Co., Ltd. Osaka Japan; ^3^ Clinical Research Department, Drug Development and Regulatory Science Division Shionogi & Co., Ltd. Osaka Japan; ^4^ Laboratory for Drug Discovery and Disease Research, Pharmaceutical Research Division Shionogi & Co., Ltd. Osaka Japan; ^5^ Project Management Department, Drug Development and Regulatory Science Division Shionogi & Co., Ltd. Osaka Japan

**Keywords:** allopregnanolone, GABA receptor, major depressive disorder, pharmacokinetics, safety

## Abstract

**Aim:**

This phase 1 study assessed the pharmacokinetics, safety, and tolerability of zuranolone in Japanese and White healthy adults, and Japanese healthy elderly subjects.

**Methods:**

This single‐center study consisted of three parts. In Part A (randomized, double‐blind), the safety, tolerability, and pharmacokinetics of single dose and 7‐day consecutive multiple doses of zuranolone 10, 20, and 30 mg and placebo were assessed in 36 Japanese adults, 24 White adults, and 12 Japanese elderly (aged 65–75 years) subjects. In Part B (randomized, open‐label, crossover), the effect of food intake on the pharmacokinetics and safety of single‐dose zuranolone 30 mg was evaluated in 12 Japanese adults. In Part C (randomized, double‐blind, crossover), the effects of single‐dose zuranolone 10 and 30 mg and placebo on electroencephalography parameters were evaluated in eight Japanese adults.

**Results:**

Single and multiple doses of zuranolone were safe and well tolerated in all subjects. Linear pharmacokinetics were observed in the studied dose range. Time to steady‐state plasma concentration was within 72 h for Japanese and White adults. Pharmacokinetic profiles were comparable between Japanese and White adults and between Japanese adults and Japanese elderly subjects. Plasma exposures of zuranolone were greater in the fed versus fasted state. Single‐dose zuranolone 30 mg increased low‐beta electroencephalography power.

**Conclusion:**

In healthy Japanese subjects, zuranolone was well tolerated; pharmacokinetic profile was unaffected by ethnicity or age; plasma exposures were greater in the fed state. The increased low‐beta electroencephalography power with the 30‐mg dose is consistent with γ‐aminobutyric acid receptor type A activation by zuranolone.

## Abstract 2

In healthy Japanese subjects, zuranolone was well tolerated; pharmacokinetic profile was unaffected by ethnicity or age; plasma exposures were greater in the fed state. The increased low‐beta electroencephalography power with the 30‐mg dose is consistent with γ‐aminobutyric acid receptor type A activation by zuranolone.

## Graphical abstract

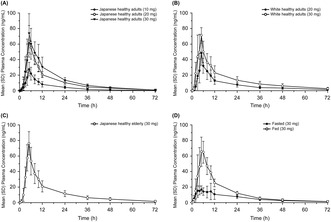

## 1 INTRODUCTION

Major depressive disorder (MDD) is a mental health condition characterized by changes in affect and function.^1^ Functional changes include cognitive, social, and occupational impairments.^1^ Approximately 322 million people worldwide suffer from depressive disorders, including MDD, which contribute to suicide deaths; an estimated 788 000 people died due to suicide in 2015.^2^ The disorder is a major problem all over the world, including Japan. In Japan, >5 million people (4.2% of the population) suffer from depressive disorders, incurring a disease burden of 850 351 life years.^2^ Every year, an estimated 20 000 people in Japan commit suicide owing to mental health disorders,^3^ commonly depression.^4^

Antidepressant medication is the mainstay of treatment for depression.^5^ In the United States (US), first‐line treatments for moderate‐to‐severe cases include selective serotonin reuptake inhibitors (SSRIs) and serotonin‐norepinephrine reuptake inhibitors (SNRIs).^5^ Despite the availability of antidepressants for MDD, an unmet treatment need remains. Current first‐line antidepressants require weeks to months of treatment before any therapeutic effect is discernible.^6^ Moreover, the risk of suicide is the highest in the first month after antidepressant treatment onset.^7^, ^8^ Approximately 30% of patients with MDD are resistant to conventional treatments.^9^, ^10^ In the US STAR*D study, which assessed the efficacy of sequential acute treatments for MDD, only 36.8% of patients achieved remission after adequate first‐line antidepressant treatment.^11^ Patients may also report poor treatment satisfaction. A US‐based study that assessed user reviews for commonly prescribed antidepressants demonstrated that low treatment satisfaction was significantly associated with emotional and behavioral adverse effects.^12^ In Japan, various antidepressants are available for depression, including tricyclic, tetracyclic, and newer antidepressants; SSRIs; SNRIs; and serotonin modulator and reuptake inhibitors.^13^, ^14^ Remission rates vary following first‐line treatment, and discontinuing or switching antidepressant therapies is common.^13^, ^15^

Antidepressants with novel mechanisms of action are being investigated as treatments for depressive disorders, including MDD. One such therapeutic agent is zuranolone, a synthetic allopregnanolone analog.^16^, ^17^ Zuranolone is currently under clinical development as an oral, once‐daily, 14‐day treatment course for MDD and postpartum depression (PPD) by Sage Therapeutic Inc. and Biogen Inc. (under the designation SAGE‐217) and for MDD by Shionogi & Co., Ltd. in Japan (under the designation S‐812217). It is a positive allosteric modulator of synaptic and extrasynaptic γ‐aminobutyric acid receptor type A (GABA_A_), a major class of inhibitory neurotransmitter receptors in the central nervous system, and a neuroactive steroid.^16^ GABA_A_ receptors govern the pathophysiology of depression, although the detailed mechanism is not necessarily clear.^17^ Allopregnanolone modulates the GABA_A_ pathway, improving depressive symptoms.^17^ Moreover, it participates in the negative feedback mechanism of the hypothalamic–pituitary–adrenal axis, the dysregulation of which through chronic exposure to psychological stress is also implicated in the etiology of mood disorders such as MDD.^18^ It is postulated that allopregnanolone demonstrates antidepressant effects by lowering the physiological impact of stress, boosting neuroprotection, and shielding against the proinflammatory immunological activation and cytokine hypersecretion associated with MDD.^19^ Allopregnanolone concentrations are low in patients with MDD, with decreased levels observed in the plasma and cerebrospinal fluid in multiple clinical and preclinical studies.^20^, ^21^ Thus, restoring neuroactive steroid and GABA_A_ function with allopregnanolone analogs, such as zuranolone, has been considered effective in treating MDD.^22^

Clinical evidence from US trials indicates that a 2‐week treatment with zuranolone has good safety and efficacy profiles in patients with MDD and PPD.^23^, ^24^ In its phase 3 study in women with PPD in the US, zuranolone administered for 2 weeks showed greater and rapid reductions in depressive symptoms compared with placebo at Day 15.^24^ Also, in its phase 2 study in individuals with moderate‐to‐severe MDD in the US, compared with placebo, zuranolone rapidly reduced depressive symptoms at Day 15^23^ and also improved patient‐reported health‐related quality of life by Day 15 after daily administration for 14 days.^25^ Brexanolone, a synthetic intravenous (IV) formulation of allopregnanolone, is the first drug approved in the US for treating PPD.^26^

Ethnicity, age, and food potentially impact pharmacokinetics (PKs) and, therefore, the efficacy or safety of a drug. We assessed the safety, tolerability, and PKs after single‐ and multiple‐dose administration of zuranolone in Japanese healthy adults (aged 20–55 years) and Japanese elderly (aged 65–75 years) and compared them with White healthy adults (aged 20–55 years). We also evaluated electroencephalography (EEG) changes in Japanese healthy adults after a single dose of zuranolone.

## 2 MATERIALS AND METHODS

### 2.1 Study design

**FIGURE 1:**
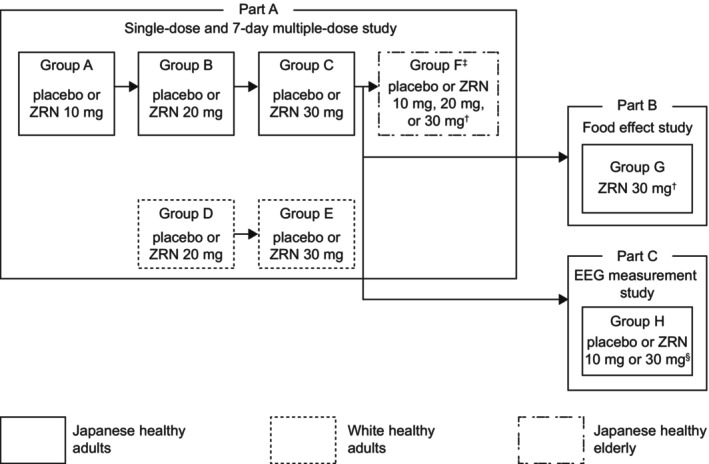
Trial design. ^†^Based on the results of Groups A–C, zuranolone 30 mg was administered. ^‡^If tolerability was not confirmed in Group F, Group F1 would be added to the study and administered a lower dose than Group F. ^§^The dose of zuranolone in Group H was defined as 10 or 30 mg. EEG, electroencephalography; mg, milligram; ZRN, zuranolone.

This phase 1 study was conducted at a single center in Japan. The trial consisted of three parts: Part A (single‐dose and 7‐day multiple‐dose study), Part B (food effect study), and Part C (EEG measurement study) (Figure 1). Part A was conducted as a randomized, double‐blind, placebo‐controlled study in Japanese healthy adults, White healthy adults, and Japanese elderly; Part B was conducted as a randomized, open‐label, 2‐way crossover study in Japanese healthy adults; Part C was conducted as a randomized, double‐blind, 3‐way crossover study in Japanese healthy adults. After eligibility was confirmed, subjects were randomized to appropriate treatment groups and documented in the study assignment table. After randomization of the study drug for Parts A and C, the study drug assignment table was sealed by the person responsible for the study drug assignment for the duration of the study period.

### 2.2 Study ethics

The protocol, protocol amendments, and other relevant documents were submitted to, reviewed, and approved by the HOUEIKAI Institutional Review Board before study initiation. The study was conducted in accordance with the consensus ethical principles derived from international guidelines, including the Declaration of Helsinki, Council for International Organizations of Medical Sciences International Ethical Guidelines, applicable International Council for Harmonisation of Technical Requirements for Pharmaceuticals for Human Use, the Ordinance on Good Clinical Practice Guidelines, and applicable laws and regulations. All subjects provided written informed consent for study participation.

### 2.3 Inclusion and exclusion criteria

Japanese and White healthy adults included male or female subjects aged 20–55 years, whereas Japanese healthy elderly included male or female subjects aged 65–75 years at the time of providing informed consent. Additionally, the subjects were required to meet the following criteria: ability to understand the study and willingness to provide written informed consent; ability to comply with all study procedures; have a body mass index (BMI) ≥18.5 to <30.0 kg/m^2^ at screening assessment; judged to be healthy by the investigator/sub‐investigator according to specific criteria, including the clinical assessment of medical history, physical examination, laboratory tests, vital signs, and 12‐lead electrocardiography (ECG) at screening and on admission, and agreement to the birth control requirements of the study.

Key reasons for exclusion included the history of clinically significant medical conditions, including psychiatric disorders; clinically significant abnormal findings during the physical and laboratory examinations at the screening visit; being at risk for suicide; history of drug or alcohol addiction/abuse, or positive drug or alcohol screen; and history of gastrointestinal surgery. The complete list of exclusion criteria is provided in Table S1.

### 2.4 Interventions

In Part A, to assess the safety, tolerability, and PKs of single‐dose zuranolone, a single 10‐, 20‐, or 30‐mg dose of zuranolone or placebo in capsule form was administered orally after a standard breakfast meal on Day 1 of the trial. To evaluate the effects of multiple doses, seven consecutive daily doses of zuranolone or placebo were administered orally on Days 4–10 of the trial after a standard dinner meal. Japanese adults were administered zuranolone 10, 20, or 30 mg; White adults were administered zuranolone 20 or 30 mg; and Japanese elderly were administered zuranolone 30 mg. In Part B, to assess the effects of food on PKs and safety, a single dose of zuranolone 30 mg was administered orally in the fast (food intake was prohibited for 10 h before and 4 h after drug administration) or fed state (30 min after a high‐fat breakfast meal) on Day 1 and Day 9 of the study in a crossover design. In Part C, to assess EEG changes, a single dose of zuranolone 10 or 30 mg was administered orally in the fed state with a standard meal on Days 1, 9, and 17 of the study. Subjects were randomized into one of three groups based on a 3 × 3 Latin square design. A single dose of zuranolone 10 or 30 mg or placebo was administered in a predetermined order depending on the assigned group.

### 2.5 Assessments

#### 2.5.1 Safety and tolerability

Safety variables included physical examination, laboratory tests (hematology, blood chemistry, and urinalysis), vital signs (blood pressure, pulse rate, respiratory rate, and body temperature), 12‐lead ECG, Columbia Suicide Severity Rating Scale (C‐SSRS), Stanford Sleepiness Scale (SSS), Modified Observer's Assessment of Alertness/Sedation Scale (MOAA/S), and Drug Effects Questionnaire‐5 (DEQ‐5). Adverse events (AEs) were classified according to system organ class and preferred terms using the Medical Dictionary for Regulatory Activities, version 21.1. AEs reported after the initial dose of the study drug were considered treatment‐emergent AEs (TEAEs). TEAEs of special interest were sedation, recorded as a TEAE in case of an SSS score ≥6 or a MOAA/S score ≤2, as well as somnolence, dizziness, and events with suspected dependency.

#### 2.5.2 Pharmacokinetics

Blood was collected to measure plasma zuranolone concentrations at predefined time points and analyzed at a centralized laboratory. For single‐dose measurements in Parts A, B (Periods 1 and 2), and C (Periods 1, 2, and 3), blood was collected predose (0 h) and 0.5, 1, 2, 3, 4, 5, 6, 8, 10, 12, 24, 36, 48, and 72 h after administration. For multiple‐dose PK assessments in Part A, blood was collected predose and 0.5, 1, 2, 3, 4, 12, and 16 h after administration on Day 4; predose on Days 5, 7, 8, and 9; and predose and 0.5, 1, 2, 3, 4, 12, 16, 24, 36, 48, and 60 h after administration on Day 10.

The following PK parameters were evaluated: maximum plasma concentration (*C*_max_), time to *C*_max_ (*T*_max_), area under the plasma concentration‐time curve (AUC), AUC extrapolated from time zero to infinity (AUC_0–inf_), AUC from time zero to the time of the last quantifiable concentration after dosing (AUC_0–last_), AUC over the dosing interval (*τ*) (AUC_0–*τ*_), terminal elimination half‐life (*t*_1/2,z_), terminal elimination rate constant (*λ*_z_), mean residence time (MRT), apparent total clearance (CL/F), and apparent volume of distribution based on the terminal elimination phase (V_z_/F).

#### 2.5.3 Electroencephalographic assessment

Changes in low‐beta EEG power (averaged absolute power at 12–25 Hz) after the administration of zuranolone and placebo were measured by normalization with baseline EEG power in frontal regions (Fz, F3, F4, and average of three electrodes). EEG measurements were obtained immediately before and 3, 5, 6, 8, and 24 h after zuranolone administration on Day 1. Enhancement of low beta‐band power was observed in the EEG assessment for the zuranolone 30‐mg group but not for the 10‐mg group, suggesting that a single 30‐mg dose is sufficient to induce GABA_A_ receptor activation in the frontal cortex via the positive allosteric modulation of synaptic and extrasynaptic GABA_A_ receptors.^27^, ^28^, ^29^, ^30^, ^31^

### 2.6 Statistical analyses

The safety analysis population comprised all subjects who received at least one dose of the study drug. The PK concentration population comprised all subjects who reported at least one evaluable plasma concentration of zuranolone. The PK parameter population included all subjects with at least one appropriately estimated PK parameter. The EEG analysis population included all subjects who received at least one dose of zuranolone or placebo and had their EEG measured immediately before administration and at least at one time point after administration.

The number of subjects and events of TEAEs and treatment‐related AEs were used to calculate the incidence of individual and overall AEs for each dose group. Laboratory tests, vital signs, ECG, C‐SSRS, SSS, MOAA/S, and DEQ‐5 results were described using summary statistics or a shift table. PK parameters were estimated using noncompartmental methods based on plasma zuranolone concentrations from all parts. For Part A, dose proportionality in Japanese healthy adults was evaluated by linear regression analysis using a power model. The dose independence in Japanese and White healthy adults, accumulation ratio in each group and PK difference at the same dose levels between Japanese and White healthy adults and between Japanese healthy adults and Japanese healthy elderly were compared using analysis of variance (ANOVA). For Part B, the effect of food on the PKs of zuranolone in Japanese healthy adults was evaluated using ANOVA, with subjects as a random effect and dietary condition (administration in the fasted or fed state), group, and dose timing as fixed effects.

The estimated changes in low‐beta EEG power from the administration of zuranolone and placebo and their 95% confidence intervals (CIs) were calculated for each time point. Specifically, for the change from baseline of amplitude, differences between zuranolone high‐dose treatment and placebo treatment and their two‐sided 95% CIs, and between zuranolone low‐dose treatment and placebo treatment and their two‐sided 95% CIs were calculated in a 3‐period by 3‐treatment crossover ANOVA model using the subject as the block factor and period and treatment drug as main effects.

No data imputation was performed during the statistical analysis, which was performed using SAS version 9.4 (SAS Institute Inc., Cary, NC, USA) and WinNonlin version 6.2.1 (Certara, Princeton, NJ, USA).

## 3 RESULTS

### 3.1 Subject disposition

Overall, 36 Japanese healthy adults (nine each in the zuranolone 10‐, 20‐, and 30‐mg groups and placebo group), 24 White healthy adults (nine each in the zuranolone 20‐ and 30‐mg groups and six in the placebo group), and 12 Japanese healthy elderly (nine in the zuranolone 30‐mg group and three in the placebo group) were enrolled in Part A of the study. Twelve and eight Japanese healthy adults were enrolled in Parts B and C, respectively. One Japanese healthy adult from the placebo group in Part A withdrew from the study; all other subjects completed the trial.

### 3.2 Subject characteristics

All subjects in the study were male, and there were no notable differences in subject characteristics between the compared experimental groups in all parts. In Part A, the mean age range for Japanese and White healthy adults was 24.7–27.1 and 28.5–30.4 years, respectively, and 69.0–70.0 years for Japanese elderly. The mean BMI range was 20.6–22.3 kg/m^2^ for Japanese healthy adults, 22.7–24.6 kg/m^2^ for White healthy adults, and 23.1–23.2 kg/m^2^ for Japanese elderly (Table 1). For subjects in Part B, the mean age was 22.9 years and mean BMI was 21.7 kg/m^2^ (Table 1). In Part C, the mean age of subjects was 23.0 years and mean BMI was 20.7 kg/m^2^ (Table 1).

**TABLE 1:** Demographic and baseline information (safety analysis population).

	Part A									Part B			Part C			
	Japanese healthy adults				White healthy adults			Japanese healthy elderly		Japanese healthy adults			Japanese healthy adults			
	10 mg	20 mg	30 mg	Pl.	20 mg	30 mg	Pl.	30 mg	Pl.	30 mg			Pl., 10 mg, 30 mg^a^	10 mg, 30 mg, Pl.^a^	30 mg, Pl., 10 mg^a^	Total
										Fasted	Fed	Total				
*N*	9	9	9	9	9	9	6	9	3	6	6	12	3	3	2	8
Age (years)																
Mean (SD)	24.7 (3.5)	27.1 (2.1)	25.2 (2.9)	25.9 (5.3)	29.7 (3.7)	30.4 (3.4)	28.5 (3.6)	69.0 (2.7)	70.0 (2.6)	23.8 (3.7)	22.0 (2.3)	22.9 (3.1)	22.3 (3.2)	21.7 (2.1)	26.0 (2.8)	23.0 (3.0)
Sex																
Male, *n* (%)	9 (100)	9 (100)	9 (100)	9 (100)	9 (100)	9 (100)	6 (100)	9 (100)	3 (100)	6 (100)	6 (100)	12 (100)	3 (100)	3 (100)	2 (100)	8 (100)
BMI (kg/m^2^)																
Mean (SD)	21.8 (1.7)	22.3 (1.9)	21.4 (1.5)	20.6 (1.2)	23.9 (2.8)	24.6 (3.1)	22.7 (1.5)	23.1 (1.3)	23.2 (0.4)	21.3 (2.3)	22.2 (0.9)	21.7 (1.7)	21.4 (1.5)	19.5 (0.6)	21.4 (1.3)	20.7 (1.4)
Drinking habits, *n*																
Yes	3	1	1	3	0	1	0	5	2	2	4	6	0	0	0	0
No	6	8	8	6	9	8	6	4	1	4	2	6	3	3	2	8

Abbreviations: BMI, body mass index; mg, milligram; *N*/*n*, number of subjects; Pl., placebo; SD, standard deviation.

^a^ 3‐way crossover design. Subjects were randomized into one of three groups based on a 3 × 3 Latin square design to receive zuranolone 10 mg, zuranolone 30 mg, or placebo.

### 3.3 Safety

In Part A, four Japanese adults reported TEAEs: one (11.1%) each in the zuranolone 10‐mg and placebo groups, and two (22.2%) in the zuranolone 30‐mg group (Table 2). In White adults, TEAEs were reported in four (44.4%), four (44.4%), and two (33.3%) subjects in the zuranolone 20‐mg, 30‐mg, and placebo groups, respectively. All TEAEs in healthy adults were deemed related to the study drug, except for that in one White adult in the zuranolone 20‐mg group. In Japanese elderly, one study drug‐related TEAE was reported in one (11.1%) subject in the zuranolone 30‐mg group. TEAEs reported in Part A included somnolence in one (11.1%) subject each in the zuranolone 30‐mg and placebo groups in Japanese adults; three (33.3%), three (33.3%), and two (33.3%) subjects in the zuranolone 20‐mg, 30‐mg, and placebo groups in White adults, respectively; and one (11.1%) subject in the zuranolone 30‐mg group in Japanese elderly (Table 2). In Part C, two (25.0%) subjects receiving zuranolone 30 mg reported dizziness, which was considered to be study‐drug related (Table 2). All TEAEs were mild to moderate and were resolved during the study period. No deaths, serious TEAEs, or TEAEs resulting in study drug discontinuation were reported. No clinically significant trends were noted in the laboratory values, vital signs, or ECG parameters. There were no findings to doubt drug preference.

**TABLE 2:** Overall summary and incidence of TEAEs (Parts A and C) (safety analysis population).

	Part A									Part C		
	Japanese healthy adults				White healthy adults			Japanese healthy elderly		Japanese healthy adults		
	10 mg	20 mg	30 mg	Pl.	20 mg	30 mg	Pl.	30 mg	Pl.	Pl., 10 mg, 30 mg^a^	10 mg, 30 mg, Pl.^a^	30 mg, Pl., 10 mg^a^
*N*	9	9	9	9	9	9	6	9	3	8	8	8
Any TEAE												
*n* (%)	1 (11.1)	0 (0.0)	2 (22.2)	1 (11.1)	4 (44.4)	4 (44.4)	2 (33.3)	1 (11.1)	0 (0.0)	0 (0.0)	2 (25.0)	0 (0.0)
Events, *n*	2	0	2	1	7	12	5	1	0	0	2	0
Incidence of specific TEAEs, *n* (%)												
Somnolence	0 (0.0)	0 (0.0)	1 (11.1)	1 (11.1)	3 (33.3)	3 (33.3)	2 (33.3)	1 (11.1)	0 (0.0)	—	—	—
Dizziness	0 (0.0)	0 (0.0)	1 (11.1)	0 (0.0)	0 (0.0)	1 (11.1)	0 (0.0)	0 (0.0)	0 (0.0)	0 (0.0)	2 (25.0)	0 (0.0)
Dysesthesia	0 (0.0)	0 (0.0)	0 (0.0)	0 (0.0)	1 (11.1)	0 (0.0)	0 (0.0)	0 (0.0)	0 (0.0)	—	—	—
Dyskinesia	0 (0.0)	0 (0.0)	0 (0.0)	0 (0.0)	0 (0.0)	1 (11.1)	0 (0.0)	0 (0.0)	0 (0.0)	—	—	—
Presyncope	0 (0.0)	0 (0.0)	0 (0.0)	0 (0.0)	1 (11.1)	0 (0.0)	0 (0.0)	0 (0.0)	0 (0.0)	—	—	—
Sinus tachycardia	0 (0.0)	0 (0.0)	0 (0.0)	0 (0.0)	0 (0.0)	0 (0.0)	1 (16.7)	0 (0.0)	0 (0.0)	—	—	—
Phlebitis	0 (0.0)	0 (0.0)	0 (0.0)	0 (0.0)	1 (11.1)	0 (0.0)	0 (0.0)	0 (0.0)	0 (0.0)	—	—	—
Diarrhea	0 (0.0)	0 (0.0)	0 (0.0)	0 (0.0)	1 (11.1)	0 (0.0)	0 (0.0)	0 (0.0)	0 (0.0)	—	—	—
ALT increase	1 (11.1)	0 (0.0)	0 (0.0)	0 (0.0)	0 (0.0)	0 (0.0)	0 (0.0)	0 (0.0)	0 (0.0)	—	—	—
AST increase	1 (11.1)	0 (0.0)	0 (0.0)	0 (0.0)	0 (0.0)	0 (0.0)	0 (0.0)	0 (0.0)	0 (0.0)	—	—	—
BP increase	0 (0.0)	0 (0.0)	0 (0.0)	0 (0.0)	0 (0.0)	0 (0.0)	1 (16.7)	0 (0.0)	0 (0.0)	—	—	—

*Note*: Adverse events were coded using MedDRA Version: 21.1.

Abbreviations: ALT, alanine aminotransferase; AST, aspartate aminotransferase; BP, blood pressure; MedDRA, Medical Dictionary for Regulatory Activities; mg, milligram; *n*, number of subjects; Pl., placebo; TEAE, treatment‐emergent adverse event.

^a^ 3‐way crossover design. Subjects were randomized into one of three groups based on a 3 × 3 Latin square design to receive zuranolone 10 mg, zuranolone 30 mg, or placebo.

### 3.4 Pharmacokinetics

**FIGURE 2:**
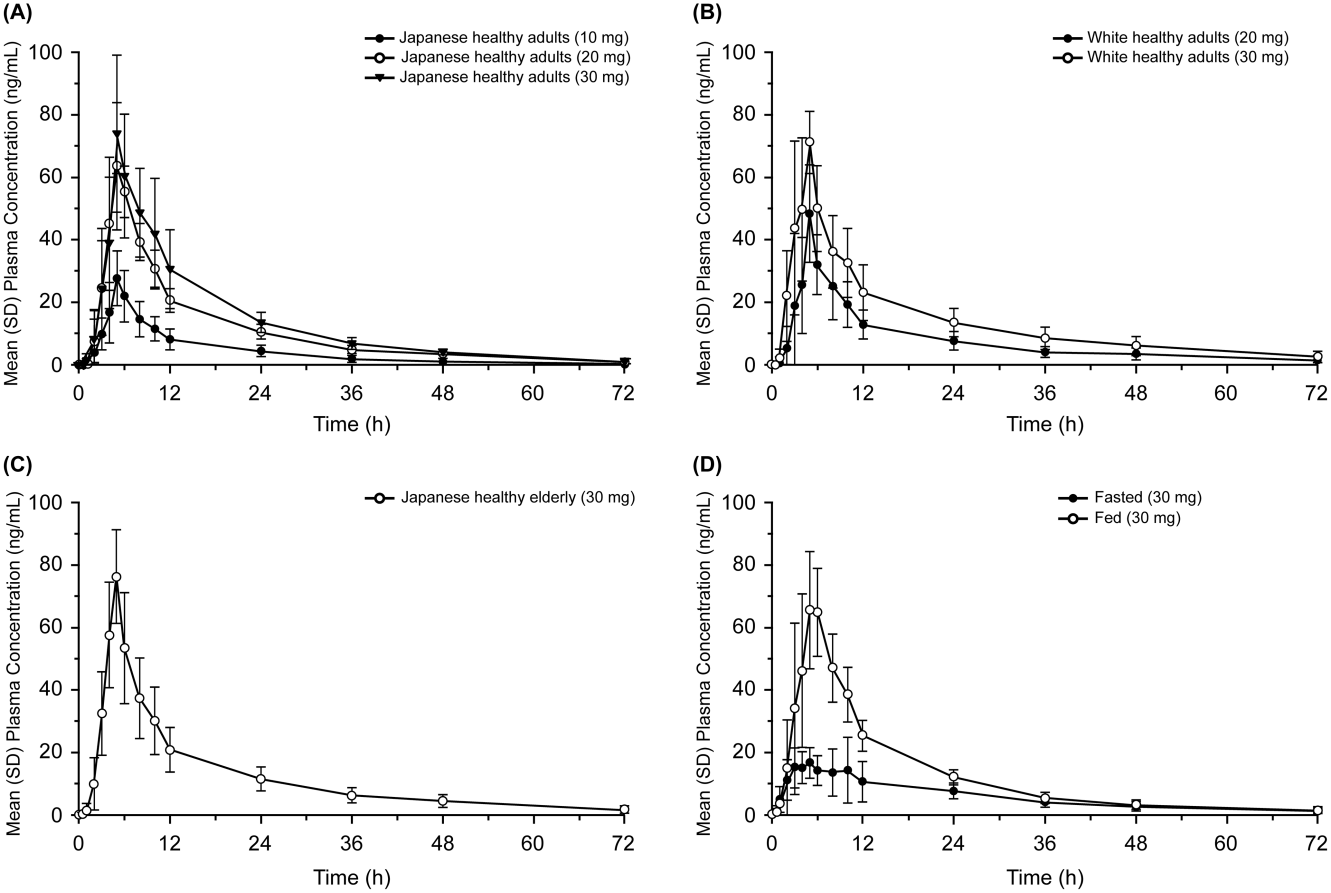
Plasma concentration profiles of a single dose of zuranolone in (A) Japanese healthy adults, (B) White healthy adults, (C) Japanese healthy elderly, and (D) Japanese healthy adults in fasted and fed states (PK concentration population). h, hour; mg, milligram; mL, milliliter; ng, nanogram; PK, pharmacokinetics; SD, standard deviation.

PK analyses were performed based on plasma zuranolone concentration data after single‐ and 7‐day multiple‐dose administration of zuranolone in Part A and single‐dose administration in Parts B and C. The mean plasma concentration profile of zuranolone after single‐dose administration in Parts A and B is shown in Figure 2. Plasma exposure levels following a single dose of zuranolone in the fed state were similar between Japanese adults (Figure 2A), White adults (Figure 2B), and Japanese elderly (Figure 2C). The median *T*_max_ of zuranolone was 5.00 h after single‐dose administration in all groups (Table 3). The geometric mean *t*_1/2,z_ of zuranolone after single‐dose administration ranged from 13.5 to 14.1 h in Japanese adults, from 18.7 to 20.2 h in White adults, and 17.9 h in Japanese elderly. *C*_max_ and AUC of zuranolone increased in a dose‐proportional manner in both Japanese (zuranolone 10–30 mg) and White (zuranolone 20 and 30 mg) adults (Table 3). The analysis comparing PK parameters (*C*_max_ and AUC) showed that following a single‐dose administration of zuranolone 30 mg, no significant differences in plasma exposures were observed between Japanese and White adults and between Japanese adults and Japanese elderly (Table S2).

**TABLE 3:** Summary of PK parameters after a single‐dose administration of zuranolone (PK parameter population).

Parameter, geometric mean (CV% geometric mean)	Part A						Part B		Part C	
	Japanese healthy adults			White healthy adults		Japanese healthy elderly	Japanese healthy adults		Japanese healthy adults	
	10 mg	20 mg	30 mg	20 mg	30 mg	30 mg	Fasted (30 mg)	Fed (30 mg)	10 mg	30 mg
Number of subjects	9	9	9	9	9	9	12	12	8	8
*C*_max_ (ng/mL)	27.6 (32.5)	67.8 (22.4)	80.3 (18.9)	50.7 (32.4)	72.7 (18.6)	76.4 (16.8)	18.3 (50.2)	74.8 (17.1)	22.4 (12.4)	55.9 (18.4)
*T*_max_ ^a^ (h)	5.00 (4.00, 6.00)	5.00 (4.00, 6.00)	5.00 (4.00, 10.00)	5.00 (3.00, 8.00)	5.00 (3.00, 5.00)	5.00 (4.00, 5.00)	4.00 (2.00, 10.00)	5.00 (3.00, 8.00)	5.60 (3.60, 5.62)	5.63 (5.60, 10.00)
AUC_0–last_ (ng · h/mL)	255.0 (43.8)	717.9 (23.5)	911.2 (17.1)	497.5 (34.7)	898.8 (32.9)	799.0 (27.6)	352.9 (38.1)	836.2 (15.1)	228.3 (16.4)	700.9 (11.1)
AUC_0–inf_ (ng · h/mL)	287.1 (39.9)	763.0 (23.3)	952.2 (16.2)	561.0 (36.9)	995.0 (33.7)	854.3 (28.2)	380.6 (35.6)^b^	873.3 (14.6)	249.9 (14.9)	735.5 (10.5)
*t*_1/2,z_ (h)	13.5 (36.9)	14.1 (29.5)	13.9 (26.6)	18.7 (51.6)	20.2 (35.6)	17.9 (24.5)	16.8 (56.2)	13.7 (36.6)	10.8 (21.7)	13.2 (39.6)
*λ*_z_ (1/h)	0.0514 (36.9)	0.0492 (29.5)	0.0498 (26.6)	0.0371 (51.6)	0.0343 (35.6)	0.0388 (24.5)	0.0413 (56.2)	0.0506 (36.6)	0.0642 (21.7)	0.0526 (39.6)
CL/F (L/h)	34.8 (39.9)	26.2 (23.3)	31.5 (16.2)	35.7 (36.9)	30.2 (33.7)	35.1 (28.2)	78.8 (35.6)^b^	34.4 (14.6)	40.0 (14.9)	40.8 (10.5)
MRT (h)	19.1 (22.7)	19.9 (24.2)	20.0 (14.5)	26.2 (39.1)	26.5 (30.7)	22.4 (19.0)	24.2 (19.3)^b^	18.3 (21.1)	18.1 (18.7)	19.7 (17.4)
V_z_/F (L)	678 (39.7)	533 (28.9)	633 (28.9)	961 (45.5)	880 (30.0)	906 (21.8)	1710 (48.1)^b^	679 (30.0)	624 (26.9)	776 (42.8)

Abbreviations: AUC_0–inf_, area under the plasma concentration‐time curve extrapolated from time zero to infinity; AUC_0–last_, area under the plasma concentration‐time curve from time zero to the time of the last quantifiable concentration after dosing; CL/F, apparent total clearance; *C*_max_, maximum plasma concentration; CV, coefficient of variation; h, hour; L, liter; max, maximum; mg, milligram; min, minimum; mL, milliliter; MRT, mean residence time; ng, nanogram; PK, pharmacokinetic; *t*_1/2,z_, terminal elimination half‐life; *T*_max_, time to maximum plasma concentration; V_z_/F, apparent volume of distribution based on the terminal phase; *λ*_z_, terminal elimination rate constant.

^a^ Median (min, max).

^b^ *N* = 11.

**FIGURE 3:**
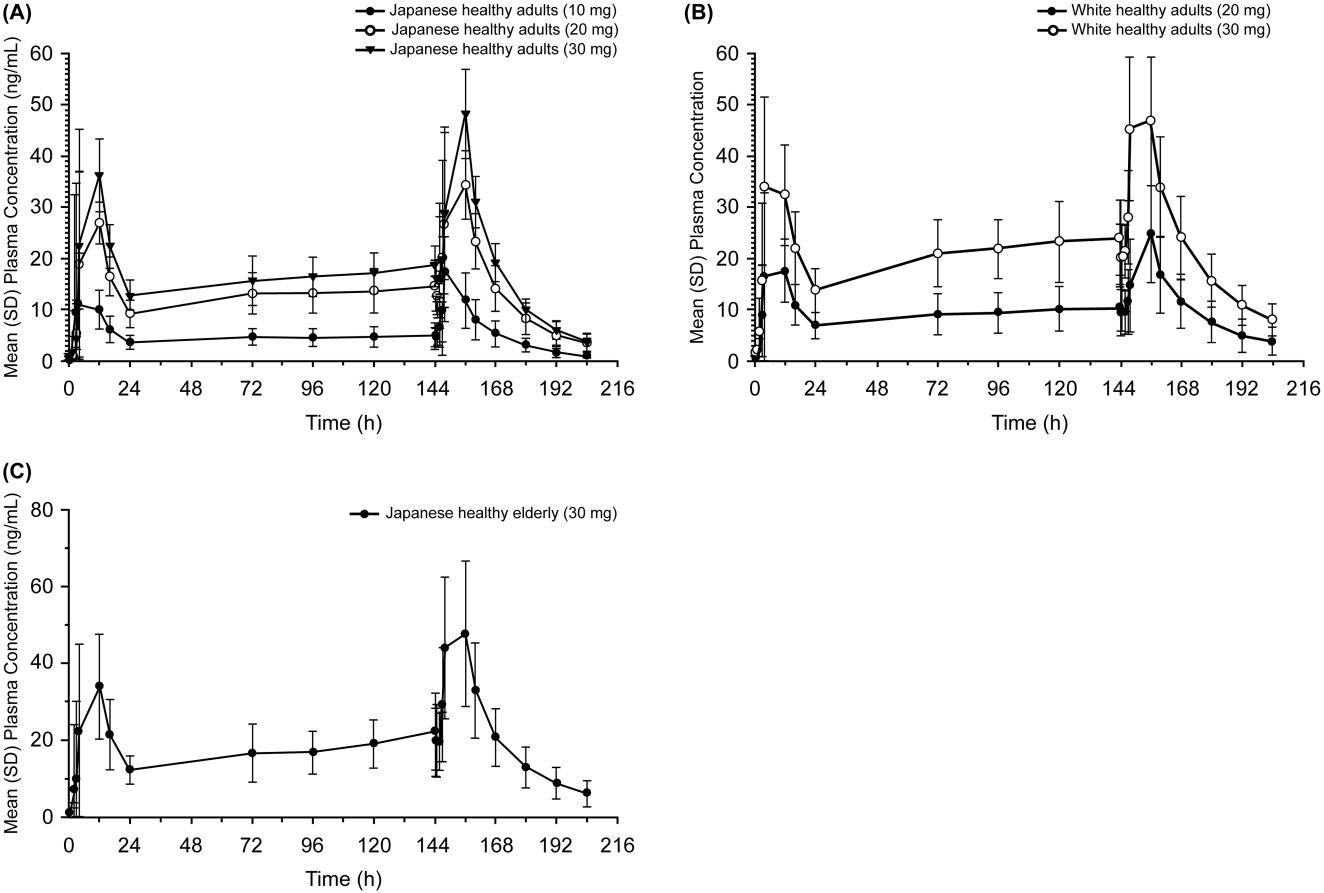
Plasma concentration profiles of multiple doses of zuranolone in (A) Japanese healthy adults, (B) White healthy adults, and (C) Japanese healthy elderly (PK concentration population). h, hour; mg, milligram; mL, milliliter; ng, nanogram; PK, pharmacokinetics; SD, standard deviation.

After multiple‐dose administration of zuranolone, plasma concentrations reached a steady state within 72 h in Japanese and White adults and within 7 days in Japanese elderly (Figure 3). For multiple‐dose administration of zuranolone, because the drug was administered after the evening meal and blood sampling was not performed during sleep, PK parameters were estimated from limited samples and could not be accurately estimated. Nonetheless, the *C*_max_ and AUC of zuranolone increased in a dose‐proportional manner in both Japanese and White adults on Days 4 (first dose) and 10 (last dose) (Table S3). At the last dose, the geometric means for *t*_1/2,z_ were 15.4–16.4 h for zuranolone groups in Japanese adults; 21.3 and 23.9 h for zuranolone 20 and 30 mg, respectively, in White adults; and 20.2 h in Japanese elderly (Table S3).

Statistical analysis of dose proportionality of *C*_max_, AUC_0–last_, and AUC_0–inf_ following single‐dose administration of zuranolone in Japanese adults showed that the 95% CIs of slope for *C*_max_, AUC_0–last_, and AUC_0–inf_ contained the value 1, suggesting that the *C*_max_ and AUC of zuranolone in Japanese healthy adults increased in a dose‐proportional manner across the studied dose range (Table S4). Parameters *t*_1/2,z_, CL/F, MRT, and V_z_/F after single‐dose administration of zuranolone and *t*_1/2,z_ after 7‐day multiple‐dose administration of zuranolone in Japanese and White adults were independent of dose across the studied dose range (Table S5).

In Part B, plasma exposures of zuranolone were greater in the fed than in the fasted state (Figure 2D). Median *T*_max_ was 4.00 and 5.00 h and the geometric mean *t*_1/2,z_ was 16.8 and 13.7 h in the fasted and fed states, respectively (Table 3). The geometric least squares mean ratios for *C*_max_, AUC_0–last_, and AUC_0–inf_ (fed/fasted) were 4.1, 2.4, and 2.3, respectively (Table S2).

The median *T*_max_ derived from plasma exposures of zuranolone found in Part C were 5.60 and 5.63 h following a single dose of zuranolone 10 and 30 mg, respectively, in the fed state. The *C*_max_ and AUC for zuranolone 30 mg were approximately 2.5 and 2.9 times higher, respectively, than those observed for zuranolone 10 mg. The geometric means of *t*_1/2,z_ were 10.8 and 13.2 h for zuranolone 10 and 30 mg, respectively (Table 3).

### 3.5 Electroencephalography

**FIGURE 4:**
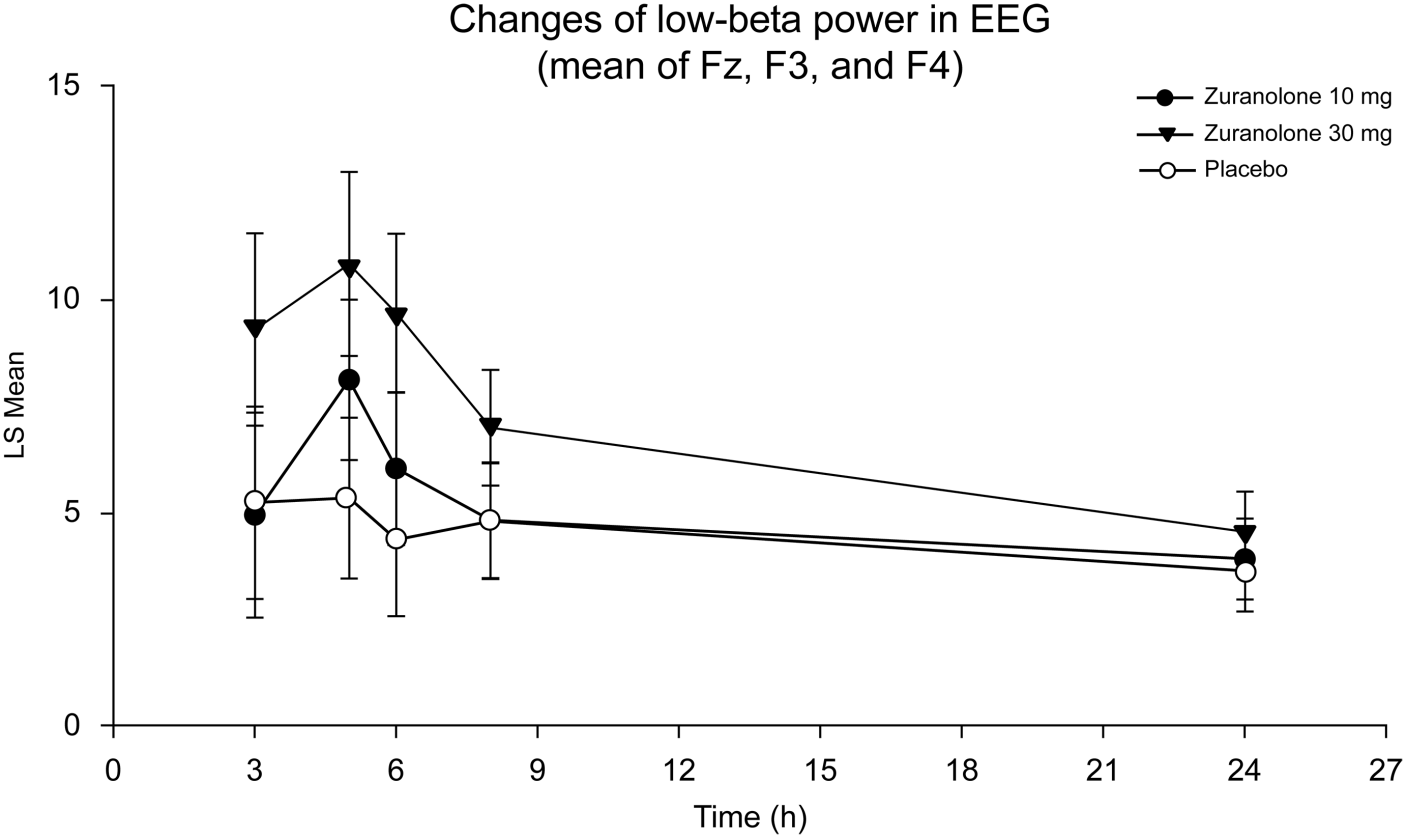
EEG power changes in the low‐beta band. EEG, electroencephalography; h, hour; LS, least squares; mg, milligram.

No substantial differences were noted in low‐beta power changes (least squares mean of Fz, F3, and F4) between the zuranolone 10‐mg and placebo groups (Figure 4, Table 4). At 5, 6, and 8 h after administration, the recorded low‐beta power showed an increase in the zuranolone 30‐mg group compared with that in the placebo group (Figure 4, Table 4).

**TABLE 4:** Low beta‐band power changes across time with zuranolone 10 and 30 mg and placebo in the Fz, F3, and F4 electrodes.

Time point (h)	Treatment	*n*	LS mean	SE	Difference from placebo (95% CI)
3	30 mg	8	9.3	2.3	4.1 (−1.5, 9.6)
	10 mg	7	4.9	2.4	−0.3 (−6.1, 5.5)
	Placebo	8	5.2	2.3	
5	30 mg	5	10.8	2.2	5.5 (1.4, 9.6)
	10 mg	8	8.1	1.9	2.8 (−0.7, 6.2)
	Placebo	8	5.3	1.9	
6	30 mg	7	9.7	1.9	5.3 (1.6, 9.0)
	10 mg	8	6.0	1.8	1.7 (−1.9, 5.2)
	Placebo	8	4.4	1.8	
8	30 mg	8	7.0	1.4	2.2 (0.8, 3.6)
	10 mg	8	4.8	1.4	0.0 (−1.4, 1.4)
	Placebo	8	4.8	1.4	
24	30 mg	8	4.5	0.9	0.9 (−0.5, 2.3)
	10 mg	8	3.9	0.9	0.3 (−1.1, 1.7)
	Placebo	8	3.6	0.9	

Abbreviations: CI, confidence interval; h, hour; LS, least squares; mg, milligram; *n*, number of subjects; SE, standard error.

## 4 DISCUSSION

Zuranolone is an oral allopregnanolone analog^16^, ^17^ that is being investigated for the treatment of MDD in Japan. This is the first study conducted in Japan to evaluate the safety, tolerability, and PKs of oral zuranolone in Japanese and White healthy adults and Japanese healthy elderly subjects. In this phase 1 study, zuranolone was well tolerated, and the PK profile was not significantly affected by ethnicity or age. The exploratory EEG analysis showed that zuranolone 30 mg enhanced the power of low beta‐band oscillatory activity.

In this study, the oral administration of zuranolone in capsule form as single (morning) and multiple doses (evening) of 10, 20, and 30 mg was generally well tolerated, regardless of ethnicity or age. The rate of TEAEs for zuranolone 30 mg was 22.2% in Japanese adults, 44.4% in White adults, and 11.1% in Japanese elderly. The most common TEAE observed was somnolence, consistent with previous studies.^23^, ^32^ In a previous US phase 1 trial, zuranolone‐related TEAEs were reported in 77.8% and 57.1% of participants in the 30‐mg 7‐day consecutive multiple‐dose morning‐ and evening‐dose groups, respectively, and common TEAEs included sedation, dizziness, and somnolence.^32^ The difference in the rate of TEAEs in both studies is most likely due to the difference in the formulation; zuranolone was administered as an oral solution in the US trial^32^ and as a capsule in the current study, likely resulting in distinctive PK profiles (reduced *C*_max_ and increased *T*_max_ with the capsule form). In another US phase 1 trial exploring the applicability of zuranolone capsules for treating insomnia, TEAEs were reported in 11.4% and 4.8% of healthy participants in the 30‐ and 45‐mg treatment groups, respectively, with the most frequent TEAEs being headache and fatigue.^33^ In a phase 2 zuranolone MDD trial conducted in the US, the most commonly reported AEs in the zuranolone 30‐mg group were headaches, followed by dizziness and nausea.^23^ In the present study, dizziness was reported in only two healthy adult subjects receiving zuranolone 30 mg.

In this study, *C*_max_ and AUC_0–inf_ of zuranolone increased in a dose‐proportional manner in Japanese (zuranolone 10–30 mg) and White (zuranolone 20 and 30 mg) healthy adults, although the exposures in Japanese adults at 20 mg were slightly higher. This confirms the proportional relationship between dose and plasma exposures that was previously established in the US phase 1 trial.^32^ Based on visual inspection of the plasma concentration profile, the time to steady‐state plasma concentration was comparable between Japanese and White adults (within 72 h) but that in Japanese elderly was longer (7 days), although no large differences in *t*_1/2,z_ were observed compared with Japanese or White healthy adults. There was no significant difference in plasma exposures of zuranolone 30 mg between Japanese and White adults, and there was no difference between Japanese adults and elderly subjects. These results suggest that the safety and efficacy of zuranolone in the Japanese population can be expected to be consistent with findings from other studies conducted on White individuals. Median *T*_max_ of zuranolone was found to be 5.00 h after single‐dose administration in all groups in Part A of the study, indicating that it is not affected by ethnicity or age and is similar for all doses in the evaluated dose range. In this study, *t*_1/2,z_ was longer (18.7–20.2 h) for White adults than for Japanese adults (13.5–14.1 h). In the previous US phase 1 study, the *T*_max_ of zuranolone was approximately 1 h with a *t*_1/2_ of 16–23 h in both single and multiple ascending doses with oral solution.^32^ The differences compared with our findings (*T*_max_ 5.00 h) are likely attributable to the difference in formulation.^32^ Food intake can affect the bioavailability of orally administered drugs.^34^ In this study, the plasma exposure of zuranolone was higher when administered orally in the fed versus fasted state, indicating a positive effect of food on bioavailability. As somnolence occurred most often after the morning dose of Day 1 and no somnolence occurred after the evening dose except in two subjects receiving zuranolone 20 mg and placebo in each, an evening dose with a meal may be appropriate for minimizing somnolence events. Therefore, in subsequent studies, zuranolone will be administered with an evening meal.^35^ Taken together, the overseas phase 1 studies have confirmed the tolerability of up to 55 mg (liquid formulation) for a single dose and up to 35 mg for multiple doses. Even if there are racial differences, the 10‐mg dose was considered to be sufficiently safe as the initial dose for Japanese patients. In addition, a 20‐mg group was added to the overseas phase 3 study and 20 mg would be the clinical dose, as pointed out by the US Food and Drug Administration. By confirming the side effects of sedation and somnolence at two doses (20 and 30 mg) in this study, we wanted to determine the optimal dose for the domestic Japanese population.

Enhancement of low beta‐band power was observed in the EEG assessment for the zuranolone 30‐mg group but not for the 10‐mg group, suggesting that a single 30‐mg dose, but not 10‐mg dose, is sufficient to induce GABA_A_ receptor activation in the frontal cortex via the positive allosteric modulation of synaptic and extrasynaptic GABA_A_ receptors. This is supported by previous clinical studies reporting that drugs with GABA_A_ receptor–positive allosteric modulation increase beta‐frequency oscillation.^27^, ^28^, ^29^, ^30^, ^31^ Our findings are also consistent with those of a previous EEG study in which zuranolone increased the power of the delta‐, theta‐, alpha‐, and beta‐frequency bands in healthy humans and of the high theta‐ and beta‐frequency bands in rats.^36^ The present EEG study demonstrated that the time course of increase of beta oscillation power by zuranolone corresponded with an increase in its plasma exposure and provided a rationale for not evaluating the 10 mg dose in the phase 2 study (Figures 2 and 4).

This study has some limitations. Only male subjects were enrolled in the study although female subjects were eligible. This is important because allopregnanolone likely plays a role in sex differences in susceptibility to brain disorders.^37^ Additionally, not all PK parameters of zuranolone in the multiple‐dose study could be appropriately estimated as zuranolone was administered after the evening meal and limited PK samples were collected.

## 5 CONCLUSION

This phase 1 study of zuranolone in Japan demonstrated that all tested doses (≤30 mg) were well tolerated and that their PK profiles were not significantly affected by ethnicity or age. Plasma exposures of zuranolone were greater in the fed than the fasted state. Zuranolone 30 mg, but not 10 mg, enhanced low‐beta power. These findings lay the groundwork for future research into the efficacy of zuranolone on depressive symptoms and its safety in the Japanese population. The recently concluded phase 2 trial of zuranolone that assessed 20‐ and 30‐mg doses in a Japanese population with MDD will shed more light on its antidepressant effects and safety in this population.

## AUTHOR CONTRIBUTIONS

TS contributed to the analysis of data and reviewing of the manuscript. DO and HY contributed to the conception and design of the study and the acquisition and analysis of data. RS, RK, and YM contributed to the conception and design of the study, analysis of data, and reviewing of the manuscript. KT and KO contributed to the conception and design of the study, interpretation of the results in Part C (EEG), and reviewing of the manuscript. TM contributed to the conception and design of the study, interpretation of results, and reviewing of the manuscript.

## FUNDING INFORMATION

This study was funded by Shionogi & Co., Ltd. Medical writing support was funded by Shionogi & Co., Ltd.

## CONFLICT OF INTEREST STATEMENT

TS, RS, RK, HY, KT, KO, and TM are full‐time employees of Shionogi & Co., Ltd and may own stock or stock options. YM and DO were full‐time employees of Shionogi & Co., Ltd. during the study and may own stock or stock options.

## ETHICS STATEMENT

Approval of the Research Protocol by an Institutional Reviewer Board: The study protocol and amendments were reviewed and approved by the HOUEIKAI Institutional Review Board prior to the study initiation.

Informed Consent: All subjects provided written informed consent prior to study participation.

Registry and the Registration No. of the Study/Trial: Not applicable.

Animal Studies: Not applicable.

## Supporting information


Tables S1–S5.

## Data Availability

The data that support the findings of this study are not publicly available due to intellectual property restrictions and informed consent not including provisions for dissemination of the data; however, the data are available from the corresponding author upon reasonable request. Furthermore, our study design had three parts: Part A was conducted as a randomized, double‐blind, placebo‐controlled study in Japanese healthy adults, White healthy adults, and Japanese elderly; Part B was conducted as a randomized, open‐label, 2‐way crossover study in Japanese healthy adults; Part C was conducted as a randomized, double‐blind, 3‐way crossover study in Japanese healthy adults. The number of patients in each treatment group is less than 10. Wherever possible data has been presented as mean (SD) or 95% CI. Also, there was no data imputation. Therefore, we do not believe that the outlier data points are of significance requiring the presentation of raw data.
