# Supplementary material for: Pharmacokinetics, safety, and tolerability of single and multiple doses of zuranolone in Japanese and White healthy subjects: A phase 1 clinical trial
Source: Neuropsychopharmacol Rep. 2023 Jun 27;43(3):346–58. doi: 10.1002/npr2.12359 (PMC10496058; doi:10.1002/npr2.12359)
Supplement: Supplementary file 1 — Tables S1–S5. [file NPR2-43-346-s001.docx]

**SUPPORTING INFORMATION**

**Table S1.** Complete list of exclusion criteria

| Reasons for exclusion included pregnancy or lactation; history of significant metabolic, endocrine, hepatic, renal, pulmonary, hematological, cardiovascular, gastrointestinal, urological, immune, neurological, or psychiatric disorders; considered ineligible for the study by the investigator/sub-investigator; history of hypersensitivity or serious treatment-related adverse events (AEs) induced by a drug; judged by the investigator/sub‑investigator as being at risk for suicide; history of drug or alcohol addiction/abuse or positive drug or alcohol screen at screening or on admission, or having undergone rehabilitation for drug or alcohol withdrawal; history of gastrointestinal surgery; recent blood donation or positive screen for syphilis, hepatitis, or human immunodeficiency virus at screening; prescribed other medications, diet, or physical therapy; history of multiple or severe allergies (except seasonal allergies); corrected QT interval (Fridericia) beyond 450 ms before study drug administration; systolic blood pressure at rest outside the range of 90–140 mmHg, diastolic blood pressure outside the range of 50–90 mmHg, or pulse rate outside the range of 40–100 beats per minute (bpm); total bilirubin, alanine aminotransferase, or aspartate aminotransferase levels >the upper limit of normal for healthy adults and >1.5x the upper limit of normal for the elderly; estimated glomerular filtration rate <90 mL/min/1.73 m^2^ for healthy adults and <60 mL/min/1.73 m^2^ for elderly subjects; use of tobacco or nicotine‑containing products within 6 months prior to admission; used prescribed or over-the-counter drugs containing vitamins, Chinese medicines, or dietary supplements (e.g., St. John’s wort) within 14 days prior to admission; used caffeine-containing products/medications within 24 hours prior to admission; consumed alcohol or alcohol-containing products within 72 hours prior to admission; consumed grapefruit, grapefruit juice, sour orange juice, orange juice, or apple juice within 7 days prior to admission; participated in strenuous exercise within 3 days before screening and admission; participated in another study or had received another study drug within 28 days prior to admission; received zuranolone; or considered ineligible for the study for other reasons by the investigator/sub‑investigator. |
| --- |

**Table S2.** Comparison of PK parameters between Japanese and White healthy adults and Japanese healthy elderly, and between fed and fasted states after single-dose administration.

| **Dose** | **Parameter** | **Geometric least-squares mean** | | | | **Ratio (90% CI)** | **Ratio (90% CI)** |
| --- | --- | --- | --- | --- | --- | --- | --- |
| **Japanese and White healthy adults and Japanese healthy elderly^a^** | | | | | | | |
|  |  | **Japanese**  **adults** | **White adults** | | **Japanese elderly** | **White adults/Japanese adults** | **Japanese elderly/Japanese adults** |
| **20 mg** | C_max_ (ng/mL) | 67.8 | 50.7 | | - | 0.7 (0.6–0.9) | - |
|  | AUC_0-last_ (ng·h/mL) | 717.9 | 497.5 | | - | 0.7 (0.5–0.9) | - |
|  | AUC_0-inf_ (ng·h/mL) | 763.0 | 561.0 | | - | 0.7 (0.6–0.9) | - |
| **30 mg** | C_max_ (ng/mL) | 80.3 | 72.7 | | 76.4 | 0.9 (0.8–1.0) | 1.0 (0.8–1.1) |
|  | AUC_0-last_ (ng·h/mL) | 911.2 | 898.8 | | 799.0 | 1.0 (0.8–1.2) | 0.9 (0.7–1.1) |
|  | AUC_0-inf_ (ng·h/mL) | 952.2 | 995.0 | | 854.3 | 1.0 (0.8–1.3) | 0.9 (0.7–1.1) |
| **Fed and fasted state in Japanese healthy adults^b^** | | | | | | | |
|  |  | **Fasted** | | **Fed** | | **Fed/Fasted ratio (90% CI)** | |
| **30 mg** | C_max_ (ng/mL) | 18.3 | | 74.8 | | 4.1 (3.4–4.9) | |
|  | AUC_0-last_ (ng·h/mL) | 352.9 | | 836.2 | | 2.4 (2.0–2.8) | |
|  | AUC_0-inf_ (ng·h/mL) | 375.0^c^ | | 873.3 | | 2.3 (2.0–2.7) | |

^a^*N* = 9 for each treatment group.

^b^*N* = 12 for each treatment group.

^c^*N* = 11 since AUC_extr_ exceeds 20%.

AUC_0-inf_, area under the plasma concentration-time curve extrapolated from time zero to infinity; AUC_0-last_, area under the plasma concentration-time curve from time zero to the time of the last quantifiable concentration after dosing; C_max_, maximum plasma concentration; CI, confidence interval; h, hour; mg, milligram; mL, milliliter; *N*, number of subjects; ng, nanogram; PK, pharmacokinetic.

**Table S3.** Summary of PK parameters after multiple-dose administration of zuranolone (PK parameter population).

| **Geometric mean (CV% Geometric mean)** | **Japanese healthy adults**  **10 mg** | **Japanese healthy adults**  **20 mg** | **Japanese healthy adults**  **30 mg** | **White healthy adults**  **20 mg** | **White healthy adults**  **30 mg** | **Japanese healthy elderly**  **30 mg** |
| --- | --- | --- | --- | --- | --- | --- |
| **Number of subjects** | 9 | 9 | 9 | 9 | 9 | 9 |
| **Day 4 (First dose)** | | | | | | |
| **C_max_ (ng/mL)** | 12.1 (63.0) | 30.8 (28.4) | 39.9 (30.6) | 21.3 (50.7) | 35.9 (32.7) | 40.0 (37.9) |
| **T_max_^a^ (h)** | 12.00 (4.00, 12.00) | 12.00 (4.00, 12.00) | 12.00 (3.00, 12.00) | 12.00 (4.00, 12.00) | 4.00 (4.00, 12.00) | 12.00 (4.00, 12.00) |
| **AUC_0-τ_ (ng·h/mL)** | 147.0 (46.7) | 368.3 (26.9) | 495.1 (30.0) | 258.5 (39.5) | 527.1 (30.7) | 464.6 (31.4) |
| **Day 10 (Last dose)** | | | | | | |
| **C_max_ (ng/mL)** | 18.9 (32.2) | 39.3 (30.8) | 48.6 (22.4) | 23.8 (35.8) | 49.9 (25.4) | 54.7 (30.0) |
| **T_max_^a^ (h)** | 4.00 (4.00, 12.00) | 12.00 (3.00, 12.00) | 12.00 (4.00, 12.00) | 12.00 (4.00, 12.00) | 12.00 (4.00, 12.00) | 4.00 (4.00, 12.00) |
| **AUC_0-τ_ (ng·h/mL)** | 227.1 (29.5) | 556.6 (24.4) | 723.0 (20.5) | 365.4 (42.6) | 837.2 (24.6) | 804.3 (28.2) |
| **t_1/2,z_ (h)** | 16.3 (27.8) | 16.4 (23.9) | 15.4 (20.8) | 21.3 (30.5) | 23.9 (37.3) | 20.2 (19.8) |
| **λ_z_ (1/h)** | 0.0425 (27.8) | 0.0422 (23.9) | 0.0451 (20.8) | 0.0326 (30.5) | 0.0290 (37.3) | 0.0343 (19.8) |

**^a^**Median (min, max).

AUC_0-τ_, area under the plasma concentration-time curve over the dosing interval (τ); C_max_, maximum plasma concentration; CV, coefficient of variation; h, hour; λ_z_, terminal elimination rate constant; max, maximum; mg, milligram; min, minimum; mL, milliliter; ng, nanogram; PK, pharmacokinetic; T_max_, time to maximum plasma concentration; t_1/2,z_, terminal elimination half-life.

**Table S4.** Dose proportionality in Japanese healthy adults after single-dose administration of zuranolone.

| **Dose range (mg)** | **Equations of PK parameters^a^** | **Lower limit of 95% CI of slope** | **Upper limit of 95% CI of slope** |
| --- | --- | --- | --- |
| **10 mg to 30 mg** | ln (C_max_) = 1.057 + 1.006 × ln (dose) | 0.773 | 1.238 |
|  | ln (AUC_0-last_) = 2.845 + 1.195 × ln (dose) | 0.926 | 1.465 |
|  | ln (AUC_0-inf_) = 3.121 + 1.126 × ln (dose) | 0.873 | 1.379 |

**^a^**ln (parameter) = Intercept + Slope × ln (dose) + Random error.

*N* = 9 for each treatment group.

AUC_0-inf_, area under the plasma concentration-time curve extrapolated from time zero to infinity; AUC_0-last_, area under the plasma concentration-time curve from time zero to the time of the last quantifiable concentration after dosing; C_max_, maximum plasma concentration; CI, confidence interval; ln, natural logarithm; mg, milligram; *N*, number of subjects; PK, pharmacokinetic.

**Table S5.** Dose independence after single- and multiple-dose administration of zuranolon**e**.

| **Parameter** | **Dose**  **(mg)** | **GLS mean^a^** | **Dose**  **(mg)** | **GLS mean^a^** | **Dose ratio** | **GLS mean ratio** | **90% CI of ratio** | |
| --- | --- | --- | --- | --- | --- | --- | --- | --- |
|  |  |  |  |  |  |  | **Lower limit** | **Upper limit** |
| **Japanese healthy adults for single dose** | | | | | | | | |
| **t_1/2,z_ (h)** | 10 | 13.5 | 20 | 14.1 | 2.00 | 1.0444 | 0.8166 | 1.3358 |
|  | 10 | 13.5 | 30 | 13.9 | 3.00 | 1.0331 | 0.8078 | 1.3214 |
|  | 20 | 14.1 | 30 | 13.9 | 1.50 | 0.9892 | 0.7734 | 1.2651 |
| **CL/F (L/h)** | 10 | 34.8 | 20 | 26.2 | 2.00 | 0.7524 | 0.6028 | 0.9392 |
|  | 10 | 34.8 | 30 | 31.5 | 3.00 | 0.9044 | 0.7246 | 1.1288 |
|  | 20 | 26.2 | 30 | 31.5 | 1.50 | 1.2019 | 0.9629 | 1.5002 |
| **MRT (h)** | 10 | 19.1 | 20 | 19.9 | 2.00 | 1.0434 | 0.8832 | 1.2326 |
|  | 10 | 19.1 | 30 | 20.0 | 3.00 | 1.0484 | 0.8874 | 1.2385 |
|  | 20 | 19.9 | 30 | 20.0 | 1.50 | 1.0048 | 0.8505 | 1.1870 |
| **V_z_/F (L)** | 10 | 678 | 20 | 533 | 2.00 | 0.7859 | 0.6073 | 1.0170 |
|  | 10 | 678 | 30 | 633 | 3.00 | 0.9343 | 0.7220 | 1.2090 |
|  | 20 | 533 | 30 | 633 | 1.50 | 1.1889 | 0.9187 | 1.5384 |
| **White healthy adults for single dose** | | | | | | | | |
| **t_1/2,z_ (h)** | 20 | 18.7 | 30 | 20.2 | 1.50 | 1.0820 | 0.7648 | 1.5306 |
| **CL/F (L/h)** | 20 | 35.7 | 30 | 30.2 | 1.50 | 0.8457 | 0.6377 | 1.1215 |
| **MRT (h)** | 20 | 26.2 | 30 | 26.5 | 1.50 | 1.0131 | 0.7652 | 1.3414 |
| **V_z_/F (L)** | 20 | 961 | 30 | 880 | 1.50 | 0.9150 | 0.6748 | 1.2409 |
| **Japanese healthy adults for multiple doses** | | | | | | | | |
| **t_1/2,z_ (h)** | 10 | 16.3 | 20 | 16.4 | 2.00 | 1.0057 | 0.8291 | 1.2198 |
|  | 10 | 16.3 | 30 | 15.4 | 3.00 | 0.9428 | 0.7773 | 1.1436 |
|  | 20 | 16.4 | 30 | 15.4 | 1.50 | 0.9375 | 0.7729 | 1.1372 |
| **White healthy adults for multiple doses** | | | | | | | | |
| **t_1/2,z_ (h)** | 20 | 21.3 | 30 | 23.9 | 1.50 | 1.1247 | 0.8563 | 1.4771 |

*N* = 9 for each treatment group.

**^a^**GLS mean presented were geometric least squares mean from ANOVA.

ANOVA, analysis of variance; CI, confidence interval; CL/F, apparent total clearance; GLS, geometric least squares; h, hour; L, liter; mg, milligram; MRT, mean residence time; t_1/2,z_, terminal elimination half-life; V_z_/F, apparent volume of distribution based on the terminal phase.
